# Evaluation of a combination “lymphocyte apoptosis model” to predict survival of sepsis patients in an intensive care unit

**DOI:** 10.1186/s12871-018-0535-3

**Published:** 2018-07-18

**Authors:** Wenqiang Jiang, Wenhong Zhong, Yiyu Deng, Chunbo Chen, Qiaosheng Wang, Maohua Zhou, Xusheng Li, Cheng Sun, Hongke Zeng

**Affiliations:** 1 The Second School of Clinical Medicine, Southern Medical University, 1063 Shatai Nan road, Guangzhou, 510515 China; 2 Department of Emergency and Critical Care Medicine, Guangdong General Hospital, Guangdong Academy of Medical Sciences, 106 Zhongshan Er Road, Guangzhou, 510080 Guangdong China; 3 Division of Laboratory, Guangdong General Hospital, Guangdong Academy of Medical Sciences, 106 Zhongshan Er Road, Guangzhou, 510080 Guangdong China

**Keywords:** Sepsis, Lymphocyte apoptosis, Immune function, Biomarker, Predictive model, Prognosis

## Abstract

**Background:**

A major challenge in sepsis intervention is unclear risk stratification. We postulated that a panel of biomarkers of lymphocyte apoptosis and immune function, termed the “lymphocyte apoptosis model,” would be an effective tool for predicting 28-day survival for sepsis patients.

**Methods:**

A total of 52 consecutive sepsis patients were enrolled. Peripheral blood samples were collected on day 1 of admission for quantification of biomarkers of lymphocyte apoptosis and immune function, including lymphocyte count, lymphocyte apoptotic percentage, expression on monocyte HLA-DR, CD4^+^/CD8^+^ T cell ratio, T helper type 1 to type 2 ratio (Th1/Th2), cytochrome c levels, and various proinflammatory cytokine levels. Sepsis severity was classified using Acute Physiology and Chronic Health Evaluation II (APACHE II) and Sequential Organ Failure Assessment (SOFA) scores. Survival was assessed at 28 days.

**Results:**

Compared with survivors, non-survivors had significantly higher lymphocyte apoptotic percentages and plasma cytochrome c levels and significantly lower lymphocyte counts, Th1/Th2 ratios, and HLA-DR expression on day 1 of admission. Multivariate analysis identified cytochrome c levels (odds ratio [OR]1.829, *p* = 0.025), lymphocyte apoptotic percentage (OR 1.103, *p* = 0.028), lymphocyte count (OR 0.150, *p* = 0.047), and HLA-DR expression (OR 0.923, *p* = 0.021) as independent predictors of 28-day mortality. A logistic regression equation incorporating the independent risk factors predicted 28-day mortality with greater accuracy than did the APACHE II score or single components biomarkers.

**Conclusions:**

The “lymphocyte apoptosis model” may be useful for risk stratification and predicting prognosis of sepsis patients.

## Background

Epidemiological data have revealed a high incidence of hospital-treated sepsis. In the United States, about 50% of patients with severe sepsis are treated in the intensive care unit (ICU). Sepsis represents 10% of all ICU admissions and is the leading cause of death in ICUs [1, 2]. Sepsis is not a homogeneous disease but, rather, a complex clinical syndrome [3]. The ambiguity of clinical findings and unclear risk stratification in sepsis have created major challenges for intervention [4]. Undoubtedly, management of sepsis patients would benefit from the ability to accurately assess their prognosis. Within this context, there is a need to identify biomarkers that address these challenges and enable timely and specific treatment [5].

The initial phase of sepsis is characterized by an intense inflammatory response [2, 6]. This phase is thought to be accompanied by downregulation of immune cell function, including that of lymphocytes, dendritic cells, and neutrophils, which could lead to immunosuppression and worsening of patient outcomes [7]. Previous studies have shown that lymphocyte apoptosis plays an important role in the stage of immunosuppression [8], suggesting that factors associated with lymphocyte apoptosis might be a potential prognostic predictors for sepsis patients.

To identify possible prognostic markers in sepsis patients, we have analyzed a number of cell types and mediators involved in lymphocyte apoptosis and immune function in the peripheral blood. We have tentatively assigned the name “lymphocyte apoptosis score model” to a combination of parameters measuring lymphocyte apoptosis (lymphocyte apoptotic percentage, lymphocyte count, and cytochrome c [cyt-c] level) and immune function (monocyte expression of human leukocyte antigen-DR [HLA-DR], T helper type 1 to type 2 cell ratio [Th1/Th2], CD4^+^ to CD8^+^ T cell ratio, and inflammatory cytokine levels). Many of these indexes of apoptotic and immune function status have been shown to be important in predicting prognosis of sepsis patients [9–13]. However, the individual biomarkers lack sufficient specificity or sensitivity to predict the clinical outcomes of sepsis patients. A similar evaluation of immune function, termed the ImmunoScore and based on enumeration of lymphocytes in the tumor core and invasive margin, had high predictive power for the postsurgical survival of gastric cancer patients [14].However, we considered that a metric based on sampling of peripheral blood could yield similarly valuable information in non-cancer patients such as those with sepsis. We hypothesized that the combined lymphocyte apoptosis and immune function parameters model (the “lymphocyte apoptosis model”) might have better specificity and/or sensitivity as a prognostic indicator for sepsis patients compared with any single biomarker.

In the present study, we aimed to evaluate the prognostic value of the “lymphocyte apoptosis model” in a well-defined cohort of sepsis patients admitted to our ICU.

## Methods

### Patients and setting

We conducted a prospective observational study by enrolling consecutive sepsis patients without multiple organ dysfunction syndrome (MODS) who were admitted to the general ICU of Guangdong General Hospital, which has 12 beds and about 700 annual admissions, from June 2014 to March 2016. The study was approved by Guangdong General Hospital Ethics Committee and was carried out in accordance with the Declaration of Helsinki. Written informed consent was obtained from all patients or their legal proxy before their enrollment in the study.Sepsis was defined as clinical evidence of infection plus at least two of the diagnostic criteria for systemic inflammatory response syndrome [15]. Patients were excluded if they were younger than 18 years old, were in end-stage of a chronic disease, and who had dysfunction of ≥2 organs within 3 days after enrollment were withdrew. All patients were evaluated in the ICU on day 1 (within 24 h after admission) and were provided conventional therapy according to the 2012 international guidelines for management of severe sepsis and septic shock [16]. The progression of sepsis and outcome at 28 days were recorded.

### Data and sample collection

Patients were followed for at least 28 days after enrollment or until death. Baseline characteristics, including demographic data, site of infection, preexisting clinical conditions, organ function, and disease severity, were recorded within 24 h after satisfying the criteria for sepsis. Disease severity was assessed using the Acute Physiology and Chronic Health Evaluation II (APACHE II) [17] and Sequential Organ Failure Assessment (SOFA) scores [18]. Blood samples were obtained in the morning of day 1. Samples were allowed to clot at 4 °C, and plasma was collected and immediately stored at − 80 °C until analysis.

### Evaluation of the lymphocyte apoptosis score and immune function status

The lymphocyte apoptosis score was calculated by measuring lymphocyte apoptotic percentage, lymphocyte count, and cyt-c levels in peripheral blood.The immune function status was evaluated by monitoring the percentage of HLA-DR–positive CD14^+^ monocytes, Th1/Th2 cell ratio, CD4^+^/CD8^+^ cell ratio, and concentrations of interleukin (IL)-6, IL-8, IL-18, interferon-γ (IFNγ), and IL-27 in plasma. All evaluations were performed on day 1 of admission to the ICU.

Plasma cyt-c and cytokine levels were quantified using enzyme-linked immunosorbent assay (ELISA) kits (R & D Systems, Minneapolis, MN) according to the manufacturer’s protocols.

Lymphocyte apoptosis was quantified using a commercially available FITC-labeled annexin V/propidium iodide kit as described previously [19]. In brief, peripheral blood mononuclear cells (PBMCs) were collected by Ficoll density gradient centrifugation, washed twice in phosphate-buffered saline (PBS), and resuspended in culture medium (RPMI 1640 plus 10% fetal calf serum) at a concentration of 1 × 10^6^/mL. Samples of 100 μL PBMCs were incubated with 500 μL 1× binding buffer, 5 μL of FITC-labeled annexin V, and 10 μL of propidium iodide (BioLegend, San Diego, CA) for 15 min.A total of 5 × 10^4^ events/sample were collected on a FACSCalibur flow cytometer (BD Biosciences, Franklin Lakes, NJ). Apoptotic cells were identified as annexin V positive and propidium iodide negative.

The CD4^+^/CD8^+^ T cell ratio and monocyte (CD14^+^) HLA-DR expression were assessed in whole peripheral blood samples collected into EDTA-containing tubes. Staining was performed within 1 h of blood collection. Cells were stained with monoclonal antibodies and isotype controls according to the manufacturer’s recommendations (all BD Pharmingen, Franklin Lakes, NJ). Aliquots of 100 μL of whole blood were incubated with FITC-labeled anti-CD4 (20 μL clone RPA-T4), PE-labeled anti-CD8 (20 μL clone HIT8a), APC-labeled anti-CD3 (20 μL clone UCHT1), FITC-labeled anti-CD14 (20 μL clone M5E2), and PE-labeled anti-HLA-DR (20 μL clone G46–6).Cells were analyzed using a FACSCalibur, and the CD4^+^/CD8^+^ cell ratio and monocyte HLA-DR levels were quantified as described by Uppal et al. and Demaret et al. [20, 21].

The ratio of Th1/Th2 cells, which has also been used as a biomarker in epidemiologic studies [22], was measured by flow cytometric detection of intracellular IFNγ (Th1) and IL-4 (Th2) after cell stimulation in vitro. Samples of 0.5 mL whole peripheral blood were collected into heparin sodium-containing tubes and mixed with 0.5 mL of serum-free RPMI 1640 medium. The cells were stimulated by addition of phorbol 12-myristate 13-acetate (25 ng/mL; Sigma-Aldrich, St Louis, MO) and ionomycin (1 μg/mL; Enzo Life Sciences, New York, NY) in the presence of brefeldin A (10 μg/mL; Sigma-Aldrich) for 4 h at 37 °C in a humidified 5% CO_2_ incubator. After stimulation, the cells were incubated with 20 μL APC-labeled anti-CD3 (clone UCHT1, BD Pharmingen) and 5 μL PerCP-Cy5.5-labeled anti-CD4 (clone RPA-T4, eBioscience, San Diego, CA) for 15 min. Erythrocytes were then lysed by addition of FACS Lysing Solution (BD Biosciences, Franklin Lakes, NJ). Cells were centrifuged, washed with 0.1% bovine serum albumin in PBS (BSA-PBS), and incubated in FACS Permeabilizing Solution (Life Technologies, Carlsbad, CA) for 10 min at room temperature. Cells were washed with 0.1% BSA-PBS and incubated with 20 μL FITC-labeled anti-IFN-γ (clone 4S.B3, BD Pharmingen) and 20 μL PE-labeled anti-IL-4 (clone 8D4–8, BD Pharmingen) for 30 min. Finally, the cells were washed with 0.1% BSA-PBS, fixed with 0.3 mL 1% paraformaldehyde, and analyzed within 12 h using a FACSCalibur. The Th1/Th2 ratio was calculated as the ratio of IFN-γ–positive to IL-4–positive CD3^+^/CD4^+^ cells.

### Statistical analysis

Statistical analyses were performed using IBM® SPSS® for Windows, version 20.0 (SPSS, Chicago, IL, USA) and R software (version 3.1.0). Statistical significance was set at *p* < 0.05. The Kolmogorov–Smirnov test was used to evaluate the normality of continuous data. For patient demographics, clinical characteristics, and components of the lymphocyte apoptosis score (lymphocyte apoptosis, lymphocyte count, and cyt-c concentration), continuous variables are presented as the mean ± standard deviation (SD) or as the median and interquartile range, depending on the distribution of the data. Unpaired *t* tests were employed to evaluate differences between two groups of normally distributed data, and the nonparametric Mann–Whitney U test was used to compare non-normally distributed data. Qualitative parameters were analyzed using a 2 × 2 contingency table and a χ^2^ test or Fisher’s exact test as appropriate. The associations between lymphocyte apoptosis and clinical severity and between lymphocyte apoptosis and other components of the lymphocyte apoptosis score were evaluated using Pearson and Spearman rank correlations. A logistic regression model was used to select the most useful prognostic factors among the components of the lymphocyte apoptosis score and immune function factors that were significantly different by univariate analysis between survivors and non-survivors at 28 days after admission. We then constructed a model based on both these lymphocyte apoptosis and immune function factors to generate a combination “lymphocyte apoptosis model” value for predicting the mortality of sepsis patients. Multivariate logistic regression analysis was applied to determine the most parsimonious combination of variables that predicted 28-day mortality. These calculations resulted in a logistic regression equation incorporating the independent risk factors*.* The prognostic performance of the independent risk factors from the multivariate logistic regression analysis, the lymphocyte apoptosis model value, and the APACHE II and SOFA scores were evaluated using area under the receiver operating characteristic (ROC) curves. ROC curves were compared using the DeLong method [23]. Patient survival was analyzed using the Cox proportional hazards model.

## Results

### Patient characteristics

**Fig. 1:**
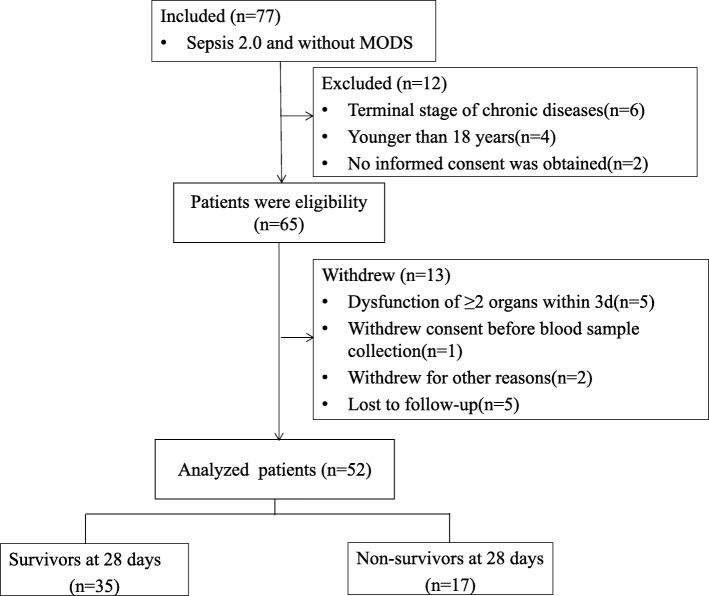
Study profile

Of the 1407 consecutive patients admitted to the ICU including 136 sepsis patients, 77 met the inclusion criteria. Of these, 25 were excluded; 15 based on the defined exclusion criteria (4 were < 18 years of age, 6 had in end-stage chronic disease, 5 had dysfunction of ≥2 organs within 3 days of enrollment), 2 failed to provide informed consent, 1 withdrew consent before blood sample collection, 2 withdrew for other reasons, and 5 were lost to follow-up. A total of 52 patients were included in and completed the study (Fig. 1). All patients were Chinese; 40 were men (76.9%) and 12 were women (23.1%). The mean (± SD) age was 62.4 ± 20.3 years. Patient demographics are shown in Table 1.

**Table 1:** Patients demographics and clinical characteristics

Variables	total (*n* = 52)	the Survival group (*n* = 35)	the non-survival group (*n* = 17)	*p*
Age (yrs), mean ± SD	62.4 ± 20.3	57.0 ± 21.8	73.3 ± 10.4	0.001
male	40(76.9)	26(74.3)	14(82.4)	0.729
APACHE II score, mean ± SD	13.2 ± 5.7	12.0 ± 5.8	15.8 ± 4.7	0.024
SOFA score, mean ± SD	4.2 ± 2.0	3.9 ± 1.8	4.8 ± 2.3	0.106
GCS score, mean ± SD	11.3 ± 3.6	11.7 ± 3.2	10.6 ± 4.2	0.290
Preexisting clinical conditions				
Diabetes	10(19.2)	9(25.7)	1(5.9)	0.137
Respiratory	15(28.8)	8(22.9)	7(41.2)	0.203
Cardiovascular	8(15.4)	5(19.2)	3(19.2)	1.000
Neurological	13(25.0)	9(25.7)	4(23.5)	1.000
Multitrauma	9(17.3)	5(14.3)	4(23.5)	0.451
Other	4(7.7)	3(8.6)	1(5.9)	1.000
Sites of infection, n (%)				
Pulmonary	26(50.0)	14(40.0)	12(70.6)	0.518
blood	6(11.5)	4(11.4)	2(11.8)	1.000
Abdomen	4(7.7)	3(8.6)	1(5.9)	1.000
thoracic cavity	3(5.8)	3(8.6)	0	–
Urinary tract	2(3.8)	2(5.7)	0	–
Soft tissue	4(7.7)	3(8.6)	1(8.6)	1.000
Biliary tract	4(7.7)	3(8.6)	1(8.6)	1.000
CNS infections	3(5.8)	3(8.6)	0	–

Values are expressed as the mean ± SD or *n* (%). *APACHE* acute physiology and chronic health evaluation, *CNS* central nervous system, *GCS* glasgow coma scale, *SD* standard deviation, *SOFA* sequential organ failure assessment

### Patient survival

Of the 52 patients enrolled, 17 died within 28 days. The causes of death were respiratory failure (*n* = 9), septic shock (*n* = 3), infection of the central nervous system (*n* = 2), liver failure (*n* = 1), and unknown causes (*n* = 2). The age and APACHE II score were significantly higher for patients in the non-survivor group compared with the survivor group (*p* < 0.001 and *p* = 0.024, respectively; Table 1).No significant differences were detected between the survivor and the non-survivor groups in gender, SOFA score, Glasgow Coma Scale score, preexisting clinical conditions, or sites of infection. A summary of the measurements is presented in Table 1.

### Quantification of components of the lymphocyte apoptosis score and immune function status in peripheral blood

As shown in Table 2, the lymphocyte apoptotic percentage, plasma cyt-c levels, and IL-18 levels were significantly higher in the non-survivor group than in the survivor group. In contrast, lymphocyte count, HLA-DR, and Th1/Th2 ratio were significantly higher in the survivors than in the non-survivors (*p* < 0.05). However, the CD4^+^/CD8^+^ ratio and plasma IL-6, IL-8, IFNγ, and IL-27 levels were not significantly different between the two groups (Table 2).

**Table 2:** Lymphocyte apoptosis score and immune function status of the survivor and non-survivor groups

Variables	the survival group (n = 35)	the non-survival group (n = 17)	*t* or *Z*	*p*
Lymphocyte apoptotic percentage (%)	20.7 ± 9.9	32.9 ± 8.9	−4.334	<0.001
Lymphocyte count (1 × 10^9^/L)	1.3 ± 0.7	0.8 ± 0.7	2.672	0.010
Cyt-c(ng/mL)	0.6(0.0–2.1)	2.0(1.2–3.4)	− 2.665	0.008
HLA-DR (%)	90.6 ± 12.7	74.9 ± 17.5	3.296	0.003
Th1/Th2	8.8 ± 4.6	6.1 ± 4.1	2.059.	0.045
CD4^+^/ CD8^+^	2.0 ± 1.4	1.6 ± 1.4	0.860	0.394
IL-6(pg/mL)	0.5(0.0–2.1)	0.3(0.0–2.7)	−0.096	0.923
IL-8(pg/mL)	4.7(0.0–5.7)	0.1(0.0–5.1)	−0.506	0.613
IFNγ (pg/mL)	35.8(19.9–157.8)	43.3(24.8–182.1)	−0.566	0.571
IL-18(pg/mL)	16.0(14.3–29.7)	22.7(17.1–56.4)	−2.365	0.018
IL-27(pg/mL)	23.5(13.8–35.8)	22.2(15.9–28.0)	0.000	1.000

Values are expressed as the mean ± SD or the median (interquartile range). Cyt-c: cytochrome c

### Multivariate analysis of components of the association between 28-day mortality and components of the lymphocyte apoptosis model

We next used multivariate analysis to determine the power of the factors significantly different by univariate analysis (age, cyt-c levels, IL-18 levels, lymphocyte apoptotic percentage, lymphocyte count, HLA-DR, Th1/Th2 ratio, and APACHE II score) to predict 28-day mortality. Data from all 52 sepsis patients were included in the final logistic regression model. Only cyt-c levels, lymphocyte apoptotic percentage, lymphocyte count, and HLA-DR expression were included as independent risk factors for 28-day mortality in the final multivariate logistic regression model (Table 3). From the logistic regression equation, the predictive value can be described by: (*p*) = exp. *Y* / (1 + exp. *Y*), where *Y* = (1.155 + [0.604 cyt-c] + [0.098 LA%]) − ([1.900 LC + 0.080 HLA-DR]), and cyt-c is the plasma cyt-c level (ng/mL), LA% is the peripheral blood lymphocyte apoptotic percentage (%), LC is the peripheral blood lymphocyte count (× 10^9^/L), and HLA-DR is the proportion of peripheral blood monocytes that are HLA-DR positive (%). Nagelkerke’s *R*^*2*^ for the lymphocyte apoptosis model was 0.695 (− 2 log-likelihood = 28.642).

**Table 3:** Multiple logistic regression analysis of risk factors for 28-day mortality among components of the lymphocyte apoptosis model

Variables	β	OR	95% CI	*p*
cytc (ng/mL)	0.604	1.829	1.080–3.097	0.025
lymphocyte apoptotic percentage (%)	0.098	1.103	1.011–1.24	0.028
lymphocyte count (1 × 10^9^/L)	−1.900	0.150	0.023–0.979	0.047
HLA-DR (%)	−0.080	0.923	0.863–0.988	0.021
Th1/Th2	0.086	1.089	0.799–1.486	0.589
IL-18(pg/mL)	−0.001	0.999	0.987–1.011	0.851
APACHE II score	−0.195	1.215	0.963–1.532	0.100
Age (yrs)	0.030	1.031	0.951–1.117	0.462
Constant	3.174			

*CI* confidence interval, *cyt-c* cytochrome c, *OR* odds ratio

### ROC curve analysis of predictors of 28-day mortality

**Fig. 2:**
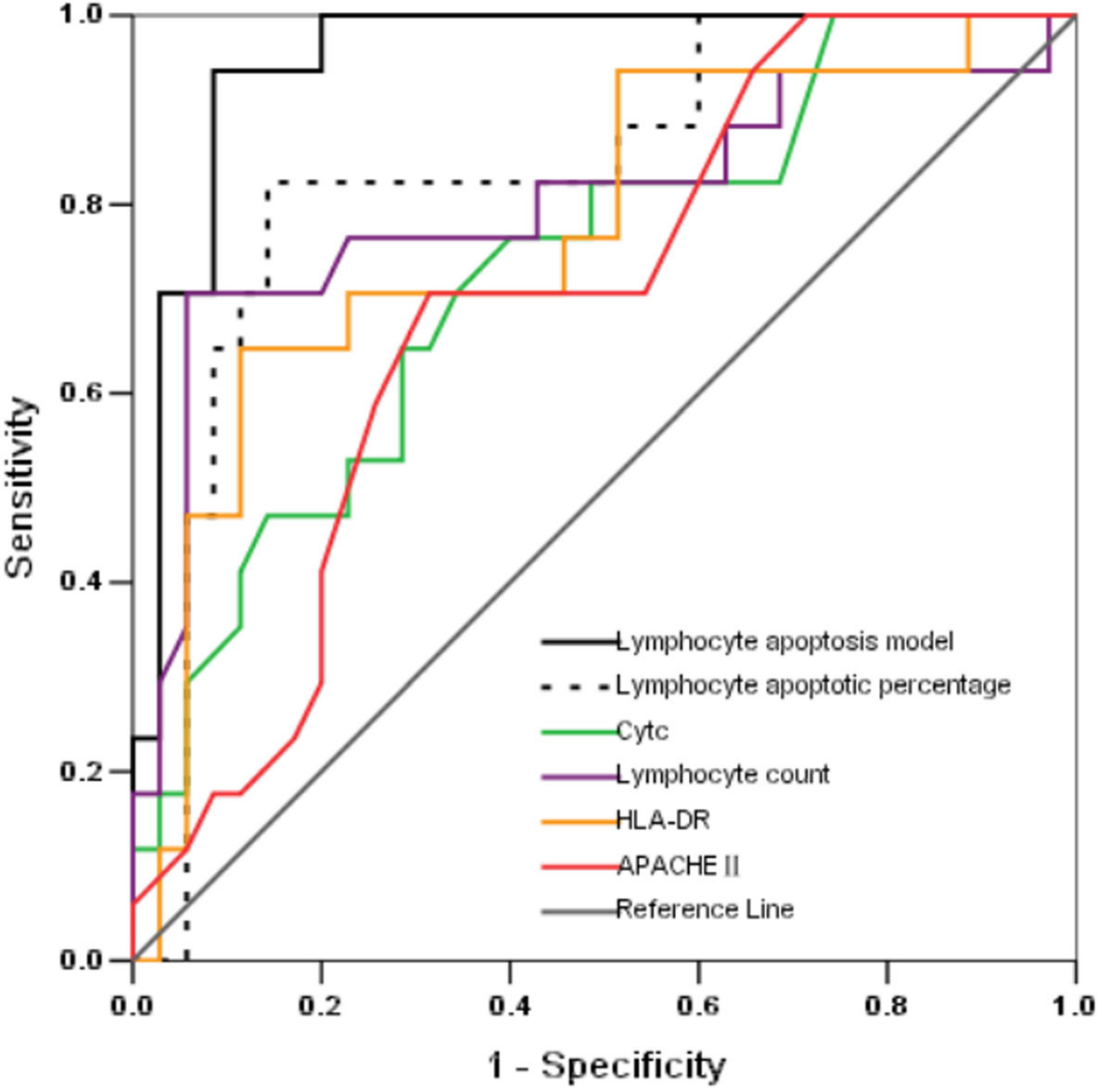
Receiver operating characteristic (ROC) curves for predicting 28-day mortality in sepsis patients. Predictive performance was evaluated for the lymphocyte apoptosis model and its individual components and the APACHE II score

We constructed ROC curves to determine the sensitivity and specificity of the lymphocyte apoptosis model and its components to predict 28-day mortality of sepsis patients. The area under the curve (AUC) for the lymphocyte apoptosis model was 0.955 (95% confidence interval [CI], 0.901–1.000), which was higher than that for the lymphocyte apoptotic percentage, cyt-c level, lymphocyte count, HLA-DR, or APACHE II score (Fig. 2). The sensitivity, specificity, and positive and negative predictive values were used to estimate the prognostic accuracy. The optimal cut-off for the lymphocyte apoptosis model predictive value was 0.286, which yielded a sensitivity and specificity of 94.1 and 91.4%, respectively, for predicting 28-day mortality. The detailed results are shown in Table 4.

**Table 4:** Value of APACHE, II, SOFA, and lymphocyte apoptosis model for predicting 28-day mortality

Variables	ROC curve			Sensitivity (%)	Specificity (%)	PPV (%)	NPV (%)
	AUC (95%*CI*)	Best cutoff	*p*				
lymphocyte apoptosis model predicted value	0.955(0.901–1.000)	0.286	<0.001	94.1	91.4	91.1	94.3
lymphocyte apoptotic percentage (%)	0.834(0.713–0.955)^a^	28.87	<0.001	82.4	85.7	73.2	90.9
cytc (ng/mL)	0.729(0.584–0.847)^a^	1.28	0.008	76.5	60.0	48.2	84.0
lymphocyte count (1 × 10^9^/L)	0.802(0.654–0.949)^a^	0.59	<0.001	94.3	70.6	60.9	96.2
HLA-DR (%)	0.773(0.632–0.914) ^a^	82.68	0.002	88.6	64.7	54.9	92.1
APACHE II score	0.696(0.550–0.842)^a^	14.5	0.023	70.6	68.6	67.0	72.1

^a^*p* < 0.05 compared with the lymphocyte apoptosis model predictive value

*APACHE* acute physiology and chronic health evaluation, *AUC* area under the curve, *CI* confidence interval, *Cyt-c* cytochrome c, *NPV* negative predictive value, *PPV* positive predictive value, *ROC* receive operating characteristic, *SOFA* sequential organ failure assessment

### Nosocomial infection, MODS and the lymphocyte apoptosis model

The lymphocyte apoptosis model predictive values were significantly higher in the patients who developed nosocomial infection during hospitalization than the others (0.54 ± 0.37 vs. 0.18 ± 0.29, *p* = 0.001) and patients who developed MODS within 7 days of admission (0.48 ± 0.41 vs. 0.14 ± 0.19, *p* < 0.001) compared with patients who did not.

### Survival and the lymphocyte apoptosis model

**Fig. 3:**
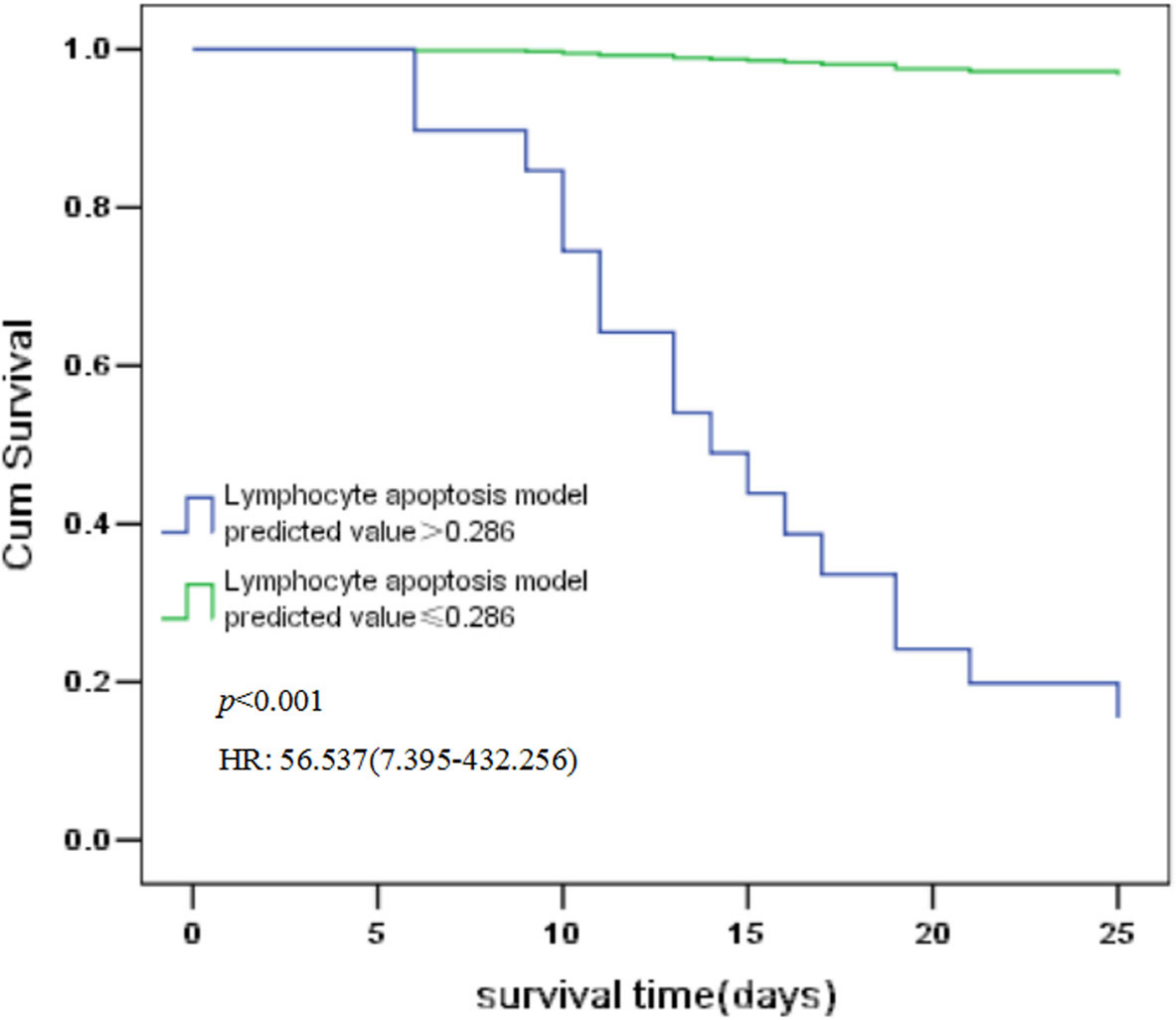
Survival curves of patients based on lymphocyte apoptosis model predictive values. Survival curves constructed using the Cox proportional hazards model show that the probability of survival at 28 days was significantly higher for sepsis patients with a lymphocyte apoptosis model predictive value < 0.286 (hazard ratio 56.537, 95% confidence interval 7.395–432.256; *p* < 0.001) than for those with a predictive value > 0.286

Using the 0.286 cut-off value for the lymphocyte apoptosis model determined by ROC curve analysis, we constructed survival curves using the Cox proportional hazards model. The analysis showed that sepsis patients with a lymphocyte apoptosis model predictive value of < 0.286 had a significantly higher probability of survival at 28 days (hazard ratio 56.537, 95% CI 7.395–432.256; *p* < 0.001) than patients with a predictive value of > 0.286 (Fig. 3).

### Clinical severity and the lymphocyte apoptosis score

We detected no significant associations between lymphocyte apoptotic percentage, plasma cyt-c level, or lymphocyte count and either the APACHE II or SOFA scores on day 1. Spearman correlation analysis showed that the lymphocyte apoptotic percentage upon admission in all patients was negatively correlated with lymphocyte count (*r* = − 0.361, *p* = 0.009) and HLA-DR (*r* = − 0.342, *p* = 0.013) but did not correlate with plasma cyt-c levels.

## Discussion

This observational study has several major findings. First, lymphocyte apoptotic percentage in the peripheral blood of sepsis patients was significantly higher in patients who died within 28 days than in patients who survived. Second, plasma cyt-c and IL-18 levels were significantly higher, and lymphocyte count, HLA-DR, and Th1/Th2 ratio were significantly lower, in the non-survivor group than the survivor group. Third, the factors significantly associated with mortality in univariate analysis were lymphocyte apoptosis percentage, plasma cyt-c levels, IL-18 levels, lymphocyte count, HLA-DR, APACHE II score and age. After stepwise logistic regression analysis of these variables, lymphocyte apoptotic percentage, plasma cyt-c levels, lymphocyte count, and HLA-DR expression were determined to be independent risk factors for 28-day mortality. Finally, the logistic regression equation to predict 28-day mortality can be described as (*p*) = exp. *Y* / (1+ exp. *Y*), where *Y* = (1.155 + [0.604 cyt-c] + [0.098 LA%]) − ([1.900 LC + 0.080 HLA-DR]). The resulting model predicted value performed better than the commonly used APACHE II score or individual model components in predicting short-term outcome in sepsis patients.

One challenge faced by intensive care physicians is how to rapidly and accurately identify sepsis patients with poor prognosis. Clearly, there is an urgent need for effective prognostic biomarkers to better inform patients and their families about potential clinical outcomes and to refine the selection of patients for inclusion in clinical trials, thereby enabling the development of novel targeted therapies.Immune dysfunction is a key factor influencing the prognosis of patients with sepsis [13, 24]. Animal studies and recent human studies have highlighted the importance of delayed sepsis-induced immunosuppression and its contribution to mortality [25]. Early and accurate prediction of prognosis allows patients to be treated with targeted immunosuppression to improve survival [26]. Immunosuppression is broadly defined as immune cell depletion and loss of immune function [27, 28]. In fact, lymphocyte apoptosis is significantly increased in the spleen and lymph nodes of sepsis patients, and this is associated with mortality. Several previous reports have indicated that lymphocyte apoptosis occurs rapidly after onset of sepsis, leads to profound and persistent lymphocyte loss, and is associated with poor outcome [10, 29]. In support of this, we found that increased lymphocyte apoptosis and decreased lymphocyte counts are both independent risk indicators for 28-day mortality.

**Fig. 4:**
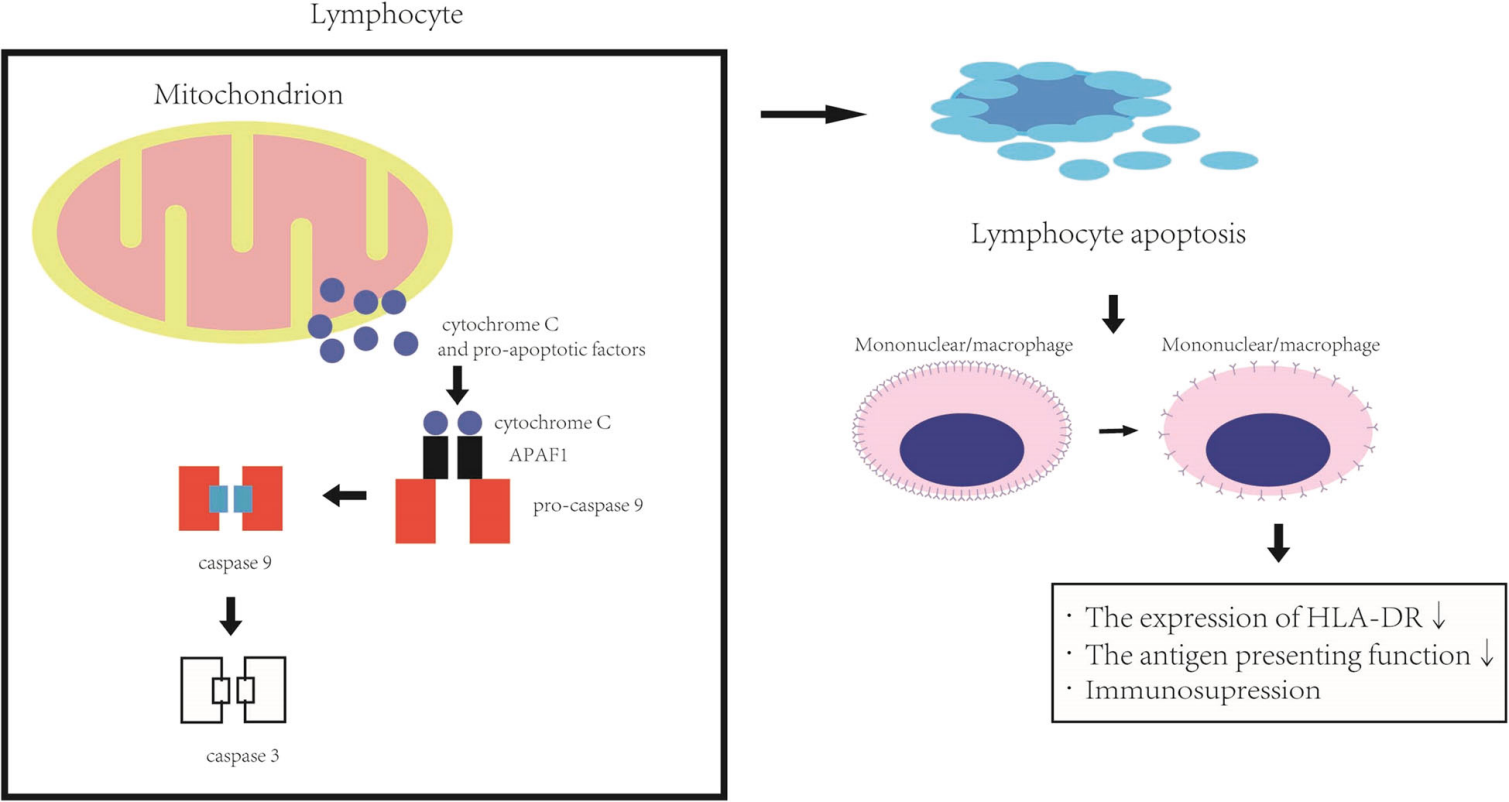
Intrinsic pathway of lymphocyte apoptosis and its effects on immune function. Cytochrome c is released from the mitochondria and, together with apoptotic protease-activating factor 1 (APAF1) and pro-caspase-9, form the apoptosome, which triggers apoptosis (intrinsic or mitochondrial pathway). In addition to lymphocyte depletion, apoptosis impairs immunity by inducing immunosuppression of the surviving cells. Monocytes/macrophages that ingest apoptotic cells show reduced HLA-DR expression, compromising their antigen-presenting function [29]

Several markers upstream and downstream of lymphocyte apoptosis may be important for predicting the prognosis of patients with sepsis (Fig. 4.) [30]. The initial triggering factors for lymphocyte apoptosis have not been fully elucidated. Hotchkiss et al. [30, 31] have reported that sepsis induces increased lymphocyte apoptosis via a mitochondrial pathway, suggesting that plasma cyt-c levels may hold promise as a prognostic marker for patients with sepsis or systemic inflammatory response syndrome [9, 32]. However, the role of lymphocyte apoptosis in the immune dysfunction observed in sepsis has recently been questioned [33–37]. Hotchkiss et al. [38] reported that increased lymphocyte apoptosis could be a component of the overall immune defect observed in septic shock. Based on our present results, we suggest that lymphocyte apoptosis is a major factor associated with 28-day mortality in sepsis patients and could be related to the immune function status. However, it is unclear how the immune function status of these patients should be evaluated. Although functional testing remains the gold standard, it is difficult to standardize. Burnham et al. [39] and Davenport et al. [40] have examined the RNA signatures of immune factors regulating antigen presentation and apoptosis, and they found that they could predict the outcome of patients with sepsis; however, the findings were not confirmed at the protein level. Our study confirms the predictive value of measuring apoptosis (lymphocyte apoptotic percentage, cyt-c levels) and antigen presentation (HLA-DR expression) at the protein level and therefore complements the earlier findings. Notably, analysis of peripheral blood components is much simpler than analysis of the transcriptomic response, since flow cytometry and ELISA can be completed within 1–2 h of sampling.

Our combination lymphocyte apoptosis model gave an AUC of 0.955 for predicting 28-day mortality, which was better than those for the component factors. Undoubtedly, this may be attributed to the assessment of multiple apoptosis and immune function status features which gives a more accurate overview of the events underlying sepsis. The various factors in the panel interact with each other in vivo and thus, as a whole, perform better than each indicator individually in evaluating the patient’s apoptosis and immune function status.

Of note, in our cohort, patients who subsequently developed nosocomial infections had significantly higher lymphocyte apoptosis model predictive values in comparison with patients who remained free of secondary infection.Similarly, high scores were closely related to the occurrence of MODS within 7 days of admission. These events may play a major role in the immune dysfunction and death observed in ICU patients, suggesting that components of the lymphocyte apoptosis model score could represent potential therapeutic targets and/or markers to guide immunotherapeutic decisions for patients with these complications.

Interestingly, we observed no correlation between lymphocyte apoptotic percentage and plasma cyt-c levels. One possible explanation for this is that plasma cyt-c is derived from apoptosis not only of lymphocytes but also of parenchymal cells. The possibility that cytosolic cyt-c might be released into the bloodstream should also be considered.

The superior predictive performance of the combinatorial lymphocyte apoptosis model is clearly illustrated by the survival curves in our study, which showed that the risk of death was significantly higher when the model predicted value was above the cut-off value.This suggests that patients with high scores should be started on immunoregulatory therapy as early as possible to prevent deterioration of their condition. Patients with high risk scores could also be evaluated at the transcriptomic level, which may identify the optimal therapeutic targets for precision treatment of individual patients.

Severity scoring systems are commonly used as classification and prognosis tools for sepsis in clinical practice. The most widely used systems include APACHE II and SOFA. However, although comprehensive, these scoring systems are subjective, cumbersome, and time consuming. Mickiewicz et al. [41] reported that a predictive model based on ^1^H nuclear magnetic resonance spectroscopy analysis of urine metabolomics was a better predictor of mortality of sepsis patients than the APACHE II and SOFA scores [42]. However, studies such as ours that evaluate a combination of lymphocyte apoptosis and immune functional status have not yet been reported. We found that our combination predictive model performed better than the APACHE II scores in predicting 28-day mortality in sepsis patients.

This preliminary study has some limitations. First, the lymphocyte apoptosis model was not tested in a validation cohort because the sample size was too small for both derivation and validation cohorts. Thus, the AUC of 0.955 may be a biased interpretation and will need to be validated in the future. Second, our patients were middle-aged to elderly. Additional studies of younger patients with severe sepsis will be needed to determine whether age affects the results. Third, a significant limitation of this study is that it was performed in a single unit. Therefore, future studies should include a comparison of the demographic and clinical characteristics of our patients and those in other ICUs.

## Conclusions

To the best of our knowledge, this is the first study describing a combination lymphocyte apoptosis model in which cyt-c levels, lymphocyte apoptotic percentage, lymphocyte count, and HLA-DR expression are identified as independent risk factors for 28-day mortality of sepsis patients. Although further testing is required, including internal and external validation cohorts, the lymphocyte apoptosis model described here could be an effective tool for predicting the prognosis of sepsis patients.

## Abbreviations

APACHE II: Acute Physiology and Chronic Health Evaluation II
CI: confidence interval
cyt-c: cytochrome c
ELISA: enzyme-linked immunosorbent assay
ICU: intensive care unit
IL: interleukin
PBMCs: peripheral blood mononuclear cells
SOFA: sequential organ failure assessment
